# The Importance of an Educational Standard

**Published:** 1921-03-12

**Authors:** 


### The Importance of an Educational Standard.

It is indisputable that men and women value most highly the prerogatives they win as the result of strenuous toil, but, should the attainment of a coveted privilege be followed by a period of mental stagnation and indifference, then apparent success may be but another name for direst failure, and the realisation of an ideal may result in the downfall of a cause. This is the position in the

Nursing Services to-day. After years of strife and bitter argument, State Registration has been achieved; the legal status of the nurse is acknowledged and approved by the nation. She has been assigned her rightful place in the web of economic activity, but, unless this is the first step towards a broader and more scientific outlook, it is the seal of failure rather than success.

The possibilities of the Nursing Services to-day are limitless. After nearly fifty years of apathy, the public begins to realise that the modern nurse is a highly skilled, responsible, efficient, well-trained professional woman. It was because the nurse herself desired it that State Registration became an accomplished fact, and it is essential that the Nursing Services themselves should realise that their future prosperity can only be assured as the result of individual ability and organised endeavour emanating from their own goodwill. When the period of grace has elapsed State Registration will be the sign of scientific efficiency or purposeless mediocrity as the nurses themselves decide. They have laid the foundations of a wonderful future, but they need courage and determination to go forward. Progress must be slow, for many of the rank and file have not yet realised their responsibilities. They have still to grasp the essential fact that amongst a democratic community it is the will of the workers that influences the decisions of the governing body and reacts upon public opinion.

If nursing is to take its rightful place amongst the professions now open to women it is essential to maintain a high ethical standard, and to inculcate amongst nurses, trained and in training, a broad professional spirit, willing and able to differentiate between personal advantage and public gain. The first step towards this desideratum is to raise the educational standard, and to do so gradually, in order that no hardship may press upon nurses already in training. There are many nurses whose lack of a well-balanced education unfits them for filling successfully administrative or teaching posts. It is for the Nursing Services themselves to decide that only women who have attained a certain educational standard shall be eligible for service in either of these capacities, and to require candidates who have no recognised educational certificates to sit for an examination on general knowledge which shall be approved by the recognised Nursing Associations. By this means women of marked ability, who during their early years have lacked educational opportunities, could if they so desired by means of post-graduate classes attain the required standard of knowledge, and would be able to realise their legitimate ambition. If this proposition were carried into effect it would unquestionably influence the decision of the General Nursing Council as to the standard of training required of a candidate before she is eligible for State Registration. No nurses' representative would be willing to fix a standard lower than that maintained by the Nursing Services themselves.

